# Biomedical Science and New Frontiers

**DOI:** 10.3389/bjbs.2022.10638

**Published:** 2022-06-20

**Authors:** Anthony Rhodes

**Affiliations:** Department of Pathology, University of Malaya, Kuala Lumpur, Malaysia

The British Journal of Biomedical Science (BJBS) has come a long way since it was first published as the Laboratory Journal now over 100 years ago, back in 1913. Its latest impact factor exceeds that of some of the most well-known pathology journals; emphasising the quality of the science now being published.

It is and always has been the official journal of the Institute of Biomedical Science (IBMS). Consequently, its articles reflect the scientific interests and specialties of IBMS members. Traditionally, the membership has been predominantly biomedical scientists employed in the health services of the UK and other countries, frequently in discipline specific areas such as Cell Sciences, Blood Sciences, Infectious Sciences, Genetics and Molecular Pathology. However, the IBMS membership profile is changing to include increasing numbers of members from the pharmaceutical and biotechnology industries, and universities, both in the UK and from overseas. The skill sets of biomedical scientists working in the health services are also rapidly changing, with an increasing number of advanced practice and consultancy roles necessary to meet the growing demands of a modernised pathology service, and with many advanced practitioners and consultant biomedical scientists now having doctoral qualifications.

The term biomedical science is used globally to describe the range of life sciences underpinning medicine. As such, biomedical research covers the whole “bench-to-bedside” journey, ranging from initial mechanistic and functional studies on cell lines, or even tiny organisms such as *Drosophila* spp. and *Caenorhabditis elegans*, through to translation of the findings to human clinical trials and predicting response to targeted therapies. Biomedical scientists in hospital laboratories are familiar with testing for established biomarkers as part of their day-to-day work. However, these established diagnostics started their embryonic life many years earlier as hypothesis driven research, subsequently tested on cell lines or animal models prior to their testing in clinical trials and eventually impacting on patient management. Set against these backdrops, the aim for the BJBS is to retain its deeply embedded foundations in the full range of disciplines that comprise biomedical science and therefore represent the interests of the many hospital-based members of the IBMS. However, in order to attract articles from the broader biomedical science communities, the journal will continue to grow by including a greater number of papers involving fundamental biomedical research that investigates the function and mechanisms of the disease process, the findings of which in years to come may be translated into new diagnostic tests that influence patient management. In short, we wish to encourage research submissions that reflect the whole of the bench-to-bedside journey.

A new feature of the journal which the Frontiers open access model supports is the introduction of Special Issues. These are a series of articles grouped on a central theme with a Guest Editor, soliciting and editing the compilation. Currently, we have a Special Issue on *Dermatopathology and Associated Laboratory Investigations in the Study of Skin Disease* edited by Dr Guy Orchard. We also have a Special Issue focusing on Women in Science which ties in with the IBMS’ celebration of 100 years of female members. We hope it will serve the community by shedding light on female researchers and provide them with a platform to showcase their work and their role in open science. With this in mind, for this Special Issue, we invite submissions from female biomedical scientists on a range of research topics related to the investigation and treatment of disease, to include but not limited to; cancer, the microbiome, cardiovascular disease and infectious disease. To complement the scientific content within the published articles we also invite authors to provide some personal reflections on life in academia that will be included within the published paper. If you wish to submit an article to either of these Special Issues or indeed to act as a Guest Editor for a new theme for which you have a passion, please do contact us at the journal, we would love to hear from you (bjbs@frontierspartnerships.org).

The BJBS has recently changed publishers from Taylor and Francis to Frontiers, which is fully open access and online. This allows the reader free access to articles published without having to subscribe to the journal, or to pay to view it. The advantage to authors is that their work can be read immediately by anybody in the world with internet access, from the moment that it is published. The value of this to the global research community was exemplified during the pandemic when most journals, even those normally requiring payment to view, made all COVID-19 related articles free to read with the aim of disseminating the results of important COVID-19 research as quickly as possible. However, as the saying goes; “there is no such thing as a free meal” and in pre- and post-COVID times, it would ordinarily fall on the authors to pay an article processing charge (APC) to publish in open access journals, and this charge can be substantial. Thankfully for IBMS members wishing to publish in the BJBS the APC is waived and members can publish their research for free.

Working closely with the Editor-in-Chief and the publishing team at the journal are four Deputy Editors who are responsible for handling the submitted articles and sourcing experts in the field to review the articles; Dr Andrew Blann, Dr Guy Orchard, Dr Mark Hajjawi and Dr Yuh Fen Pung. In addition, the editorial board members are appointed based on their expertise in different areas of biomedical science and their experience in reviewing papers. As the journal continues to grow, we will expand the editorial board to provide an increased range of expertise.

In March 2022, the IBMS held its biennial conference at the International Convention Centre, in Birmingham, UK with over 3,000 delegates in attendance. In addition to the main programme, biomedical scientists presented over 80 posters or short communications on a range of research topics. In order to capture and record the valuable contributions being made by IBMS members, the journal will work with Frontiers to publish all accepted abstracts in an e-book to be accessed via the journal’s website. This will commence with the next IBMS Congress in 2023.

In summary the BJBS, working in symbiosis with the IBMS, aims to deliver a quality publication that reflects the growth and impact of biomedical scientists and biomedical research on World health. In order to achieve this aim we greatly look forward to working with you and in receiving articles from across the globe.

